# Pediatrics

**Published:** 1898-07

**Authors:** Maud Josephine Frye

**Affiliations:** Physician to the babies’ ward, Erie County Hospital


### Progress in Medical Science.

### PEDIATRICS.

CONDUCTED BY MAUD JOSEPHINE FRYE, M. D.,
Physician to the babies' ward, Erie County Hospital.

### UNSTERILISED MILK AND THE DEATH-RATE.

AMONG the papers read before the national conference of mayors and councilmen, held at Columbus, Ohio, during September, 1897, was one on The influence of a pure milk supply on the death-rate of children, by Nathan Straus, of New York, the founder of the depots for the supply of sterilised milk to the poor. Mr. Straus said, in part, as reported by the *Journal of the American Medical Association:* There is practically no milk delivered for general consumption in cities that is fit to be fed in its natural state to young children. I think I have fairly demonstrated the proposition that many thousands of infant lives are annually sacrificed by the neglect to supply for the nutriment of children milk which has been sterilised. I hold that neglect to be criminal and I leave it to you to fix the responsibility for it. We punish murder with the penalty of death, and yet we allow murder to be committed by the wholesale in every populous community of this land with no thought of its punishment and little thought of its prevention.—*Archives of Pediatrics.*

---

### MUNICIPAL CONTROL OF MILK SUPPLY.

HEATH (*Transactions of Med. Soc. of State of New York*) in considering the subject of municipal control of the milk supply suggests that municipal ordinances should embody the following features :

1. All milk houses should be of sufficient and proper size and should have light and ventilation, complying with the rules of hygiene pertaining thereto.

2. They should be constructed of such material as to be sanitary and as much as possible of nonabsorbent material, or so made as to prevent frequent and effectual flushing and cleansing.

3. Communication by doors or other openings, with water-closets, sleeping apartments, or other rooms liable to become a source of offense, should be strictly prohibited, nor should milk-rooms be used for any other purpose whatever than the storage of milk.

4. Storage and cooling boxes should be substantially constructed, lined with metal, elevated well above the floors and away from surrounding walls, to permit thorough cleaning. They should be ventilated into the outer air and properly drained, but no drainage from them should directly communicate with the drainage or plumbing.

5. All plumbing should be strictly sanitary.

6. Rules with specific detailed directions for periodical cleaning of milk-house, storage boxes, cans, bottles, utensils and information regarding the care of milk and possible source of contamination should be adopted and conspicuously posted in the milk-room.

7. Cans should be only of such depth and such construction as to permit ready and perfect cleaning and it should be obligatory that they be cleansed before being returned to the dairy.

8. It should be obligatory that each and every can be tagged at its source, stating the character of the milk, its source, specific gravity as determined at the dairy, with the date and hour of shipment.

9. Milk-rooms should be permitted in cellars only under exceptional circumstances, and then should comply with every requirement of modern hygiene.

10. No milk-house or milk-room should be under the same roof or in connection with any horse or cow stable, nor should it be connected with any unsanitary buildings or surroundings.

11. Construction, equipment, care and management should be such as to obviate the use of disinfectants and their presence or use should be prohibited. Any odor or evidence of them about a milk-house should be construed as evidence of defective sanitation.

12. During the summer months no milk should be used when over twenty-four hours old, and during the winter the age of the milk should not exceed thirty-six hours.

13. Milk on the wagon should be thoroughly protected from the weather, and especially during the summer months, by being properly iced, and should be delivered within certain prescribed hours.

14. No milkman should deliver bottles containing milk, nor leave any receptacle of his own containing milk, at any house placarded as containing a contagious disease, nor should he receive any empty bottles or receptacles from such house, or be permitted under any circumstances to fill or refill any bottle while on the wagon.

A record of all cases of acute contagious diseases occurring upon the route or in the service of each milkman should be kept. Daily reference and scrutiny of the same should be made and upon suspicion of relationship of such contagious disease to any one source of the milk-supply, or any one milk route, immediate investigation and action should follow.

The dealing in milk in small quantities by small shopkeepers, such as grocers, confectioners and others, should be abolished.

---

### HEAT A FACTOR IN THE PRODUCTION OF SUMMER DIARRHEA.

COHN (*Arch. für Kinderheilk.*) studied the milk, the bottles and the artificial food of a number of infants during several years and finds that even where the food is in good condition and unchanged as in breast-fed babies, the heat of summer may disturb the digestion of infants in some unknown way and this must be considered as a factor in the etiology of diarrhea in artificially fed children during the summer months.—*Am. Jour. of Obstetrics.*

THE PHARMACIST AS A MILK DEALER.

In a recent address to the students of the Philadelphia college of pharmacy, Dr. J. Madison Taylor, professor of diseases of children in the Philadelphia polyclinic, urges that in view of their special training, pharmacists prepare and offer for sale sterilised cow's milk and the different modifications of milk.—*Medical Review of Reviews.*

